# 

**Published:** 1805-06-01

**Authors:** 


### To CORRESPONDENTS.

Mr. Cabani's Work, entitled, "Coup d'OEil sur les Revolutions & sur la Reforme de la Medicine," will be reviewed in our next Number.

Communications are received from Mr. Cribb, Mr. Pulley, Mr. Simmons, Mr. Bishop, Dr. Kinglake, and Medicus.

---

END OF VOL. XIII.

### INDEX.

### A.

ABERDEENSHIRE, on the difeafes of 28
Abernethy's, Mr. lectures announced 95
Abfcefs of the cheft, cafe of 21
Academicus, on dyfodontophyfis 515
Achillis, cafe of a ruptured tendo 494
Acid, on the ufe of nitrous, in diabetes mellitus 153
—— on fulphuric 110
Acids, to preferve copper veffels from the action of 288
—— on mineral 112
Adams, Dr. on vaccination 1
—— Mr. Ring's reply to 168
—— on the flough 6, 400
—— Dr. succeeds Dr. Woodville at the fmall-pox hofpital 575
Adipofe farcoma, on the 388
Ægineta, mentions the ligature 392
Æriform fluids, on 109
Affufion, on cold, in colic 18
Africa, on the towns in 118
Agues, on white vitriol in 138
Aikin's, Meffrs. dictionary of chemiftry announced 192
Alcarez, Dr. on the ufe of oil in yellow fever 17
Alcohol, on 220
Allen's, Mr. lectures announced 94
Ammoniac, on fal, in vinegar 497
Amphibia, on the heart of 430, *note*
Amphibious animals, on 370
Amputation, on 96
Amufements, utility of public 115
Anderfon's, Dr. letters on vaccination in India 283, 381
Anglicanus, Fracaftorius on the fudor 209
Animal body, fource of oxygen in the 107
Animals endued with inherent electricity 85
Anfelmi's, Dr. curious fact in natural hiftory 287
Antimony, on muriat of 447
—— on fub-muriate of 225
Aphtha, on 298
Apoplexy allied to gout 146
Armenia, whether the fyphilis was originally brought from 89
Armiger's, Mr. demonftrations announced 95
Army, on the medical department of the 275
Arfenic, on folution of, in chronic rheumatifm 16, 525
Arteries, on fecuring the 101
Afcites, cafe of 303
Afphyxia, efficacy of galvanifm in 375
Affes frequently die at the foot of Vefuvius 109
Afthenia, cafes of fimple 476
Athletic exercifes, on training perfons for 532
Atmofphere, its influence in epidemics 210
Averno on the Lago de 109
—— its connection with difeafes 105
—— on the proper ftate of for health 106
—— Sydenham on the 209
Atmofpheric difeafes, on 467

### B.

Babington's, Dr. lectures announced 94
Baddam's, Dr. lectures announced 96
Baker's cafe, on Mr. 144
Balfam of fulphur, compofition of 502, *note*
Bardfley, Dr. on hydrophobia 155
Bark, on its efficacy in gout 350
Bartholomew hofpital, lectures at 95
Baths, warm, of Ruffia, on the 77
—— on vapour 522
Batty's, Dr. lectures announced 96, 288
—— letter from Dr. Lind to 574
Bayntun's plafter, its ufe in tinea capitis 8
Beds, on burning thofe of deceafed perfons 115
Bell's, Mr. propofal for a mufeum at Edinburgh 191
Bengal, ftate of vaccination in 336
Beft, Mr. on uterine rupture 234
Bethune, Mr. on inflammatory affections 549
Bill, Mr. on tinea capitis 9
Biot, M. forms water from hydrogen and oxygen 575
Blackly's, Dr. recipe for dropfy 289
Black-vomit, experiments on 378
Bladder, on the puncture of the urinary 18
Blair's, Mr. queries on fyphilis 80
—— lectures announced 96
Bleeding in fever, on 277
Blifters in lepra, on 140
Blood-letting in dyfentery, on 562
Boils produced by fixed air 36
Bond's, Mr. cafe of reinoculation 38
Bone, on divifion of the, in amputation 100
BOOKS, CRITICAL ANALYSIS OF MEDICAL.—Enquiry into the Efficacy of

Oxygen in the Cure of Syphilis, by Charles Platt, 78. The Invalid, with the Means of enjoying Health and Long Life, by a Nonagenarian, 81. Elements of Galvanifm, by C. H. Wilkinfon, 82, 371. Practical Treatife on Efficacy of Cowhage, by W. Chamberlaine, 87. The Edinburgh Medical and Surgical Journal, 177. Some recent Cafes of Small Pox fubfequent to Vaccination, by W. Goldfon, 268. Plan to prevent Malignant Contagion, by R. Pearfon, M. D 272. Account of Two Cafes of Gout, by A. Edlin, 274. On the Medical Department of the Britifh Army, by R. Jackfon, M.D. 275,472,564. Obfervations on Cancer, by E. Home, 361. Account of a Painful Affection of the Nerves of the Face, commonly called Tic Douloureux, by S. Fothergill, M D. 367. Popular Compendium of Anatomy, by W. Burke, 370. Reply to Mr. Edlin's Two Cafes of Gout, by R. Kinglake, M. D. 461. Obfervations on the Cow Pox, by R. Squirrell, M D. 465. Treatife on Lues Bovilla, or Cow Pox, by B. Mofeley, M. D. 466. Examination of the Evidence relative to the Cow Pox by W. R. Rogers, 468. A Treatife on Febrile Difeafes, by A. P. Wilfon, M. D. 554. A General Treatife on Cattle, by John Lawrence, 564. A Botanical Dictionary, by Colin Milne, LL. D. *ib.*

BOOKS, NEW MEDICAL ANNOUNCED. Mr. Blair's Edition of Dr. Girtanner on the Venereal Difeafe, 89. Sir John Sinclair's Code of Health and Longevity, 192. Meff. A. C Aikin's Dictionary of Chemiftry, *ib.* Dr. Clutterbuck's Inquiry into the Seat and Nature of Fever, *ib.* Dr. Stock's Collections on the Effects of Cold as a Remedy, *ib.* Dr. Thornton's Anfwer to Objections againft Vaccination, *ib.* Dr. Rufh's Elements of Life, 288. Dr. Rickerton's Treatife on the Means of Preferving Health and Preventing Difeafes, *ib.* Mr Young's Treatife on Cancer, 383. Mr. Rogers's Examination of Evidence on Cow Pox, *ib.* Dr. Reid's Treatife on Confumption, 479. Dr. Harty's Obfervations on the Simple Dyfentery, 576. Dr. Jackfon on the Difeafes which prevailed at Gibraltar, *ib.* Mr. Parkinfon on Gout, *ib.*

Botany, on the ardent purfuit of 5
Botany Bay, on the fmall pox and cow pox at 346, 7
Bradley's, Dr. lectures announced 96
Britifh oil, compofition of 502
Bronchocele, on the 13
Brookes's, Mr. lectures announced 96
Brugnatelli on galvanifm 373
Bruifes, effects of fal ammoniac and vinegar in 508
Bubo, efficacy of fulphat of foda in 38
——— on 435
Bunofus, cafe of 399
Burn, cafe of 14

C.

Canals, advantages of 117
Cancer, on open 294
——— Mr. Young's Treatife on, announced 383
——— review of Mr. Home's obfervations on 361
Carbonic acid gas, on, as productive of difeafe 109
Cantharides internally adminiftered in hydrophobia 159, *note*
Cafes, on the publication of medical 207
Catalepfy, cafe of 180
Catarrh, on 209
Catfkill, account of the yellow fever at 304
Celfus, his account of the ligature 392
Ceylon, progrefs of vaccination in 122
Chamberlaine's, Mr. treatife on cowhage in worms, reviewed 87
Chancre, on 80
Chaptal's letter on vaccine inoculation 419
Cheft, cafe of abfcefs in the 21
Chevalier's, Mr. lectures announced 96
Chenevix, Mr. on his powder 225
Chicken pox, on the 5, 58, *note*
Children, on the teething of 516
China, on the influenza in 487
Chorea fancti viti, obfervations on 401
——— on the treatment of, by purgatives 178
Chriftie, Mr. on vaccination 122
Churches ought to be fumigated 115
——— impropriety of burying in 116
Clement, Mr. on herpetic eruptions 415
Clergy, on the influence of the 116
Cline's Mr. lectures announced 94
Clutterbuck's enquiry on fever announced 192
Cocks, on training game 537
Coates's, Mr. cafe of a female child 179
Cobra de capello, on the bite of the 153, *note*
Coleman's, Mr. lectures announced 94
Colic, on cold affufion in 18
——— frequent in the Highlands 36
Contagion, review of Dr. Pearfon's plan to prevent 272
——— on the origin of 210
——— queries on preventing 190
Confumption, cafe of 323
Contufion, general treatment of 385
Cooper's Mr. lectures announced 94
Copper veffels, how to preferve from acid 288
——— leaf, effect of galvanifm on 372
Correfpondents, to, 96, 192, 288, 383, 480, 576
Coup de foleil, on 487
Cow fucked by a fnake 287
Cowhage, on its efficacy as a vermifuge 87

Cow-pock institution at Dublin, account of 379

Cow pox, Dr. Adams on 1

——— at Botany Bay, on the 347

——— see VACCINATION

Cox's, Dr. observations on chorea sancti viti 401

——— on vaccination 40

Cranium, on fractures of the 526

Crawford's theory of respiration imperfect 373

Creaser's, Mr. letter to Dr. Walker 186

Crowfoot's, Mr. case of burn 14

Cullen, Dr. defence of 141

——— on dyspepsia 207

Cuming, Mr. on mercury in venereal affections 395

——— Mr. Wilkinson, in reply to 135

——— his answer to ditto 325

Cupping in hydrophobia, on 242

Curry's, Dr. lectures announced 94

Cutaneous eruptions, on 537

Cynanche parotidea, on sal ammoniac as a remedy for 509

Cynanche trachealis, on 555

D.

Davies, Mr. on puncture of the urinary bladder 10

——— on the bronchocele 12

Dawson Mr. on procidentia uteri 129

——— on mercury, in venereal affections 549

Deafness, on 451, *note*

Death, galvanism the criterion of 86

De Cairo, Dr. letters to 122

Dennison's Dr. lectures announced 95

Devonshire colic, on the 21

Diabetes mellitus, case of 152

Diseases in an eastern district of London, account of, from Nov. 20 to Dec. 20, 1804 89

——— from Dec. 20, 1804, to Jan. 20, 1805 186

——— from Jan. 20 to Feb. 20 282

——— of the highlanders, on the 28

——— in the Finsbury Dispensary 375, 476 567

——— on the connexion of the atmosphere with 105

——— of hot climates, on the 481

Dissection, case of 490

Dobbyn, Mr. on the rabies in dogs 478

Dogs, origin of the rabies in 478

——— receipt for the bite of mad 297

——— frequently die at the foot of Mount Vesuvius 109

Domeier, Dr, on the epidemical fever in Spain 103

Douglas, Dr. on the rupture of the gravid uterus 237

Drew's, Mr. cases of tumours in the pelvis 178

Dropsy, efficacious remedy in 288

Dublin, report of the cow-pock institution at 379

——— mortality of horses at 216, *note*

Duncan, Dr. on mal-formation of the urinary organs 179

Dunning, Mr. on vaccination 417

——— in reply to Dr. Walker 542

Dwight, Dr. on the yellow fever at Catskill 304

Dysentery, on blood-letting in 562

Dysodontophysis, case of 515

E.

Ear, proposed establishment for diseases of the 96

Earnest's, Mr. case of diabetes mellitus 152

Earthquakes, frequency of 110

Edinburgh Medical and Surgical Journal reviewed 177

——— proposal for an anatomical museum at 191

——— on the pharmacopæia of 219

Edlin's, Mr. case respecting cold water in gout, reviewed 274

——— remarks on 143

——— Mr. Martal on 144

——— Dr. Kinglake's reply to 460

Edwards's, Dr. lectures announced 95

Egypt, how to prevent the plague in 117

Electrical machine, account of a new 28

Electricity, on animal 85

Enteritis, case of 182

Epidemic in Spain, on the origin of 103

——— proposed methods for preventing 114

——— how to prevent its return 116

Eruptions, on herpetic 415, 541

——— observations on vaccine 2

——— on the effect of cutaneous 537

——— Dr. Jenner on herpetic 541

Erythema mercuriale, cases of 177

Essence of mustard, composition of 502

Excoriation of the glans and prepuce on the 386

Experiments galvanic 84, 372

——— on platina 90, 288

——— on black vomit 378

——— on muriat of antimony 447

Eye, proposed establishment for diseases of the 96

F.

Febrile contagion, on 209

——— affections, on 376

——— diseases, on 554

Female child, singular case of a 575

Females in the highlands, description of the 29

Ferguson, Dr. on the contagion of fever 37

——— on leeches 299

——— his case of ascites 303

Fever, on the contagion of 37

Fever, on the treatment of 276, 472, 564
——— on the Spanifh epidemical 103
——— queries to prevent contagious 190
——— on that at Gibraltar 193
——— account of that at Catfkill in America 304
——— cafes of, in the military hofpital at Canterbury 348
Fevers not frequent in the Highlands of Scotland 37
Ffirth, Dr. on black vomit 378
Fife, Mr. on inoculation 63
Finfbury Difpenfary, accounts of difeafes in the 375, 476, 567
Fire, method of preferving inflammable bodies from 288
——— on fubterraneous 108
Fifhes, on the refpiration of 371
Fixed air produces boils 36
Flap operation, on the 98
Fogo's, Mr. anfwer to Dr. Trotter, 207
Forbes, Mr. on inoculation 517
Fothergill, Dr. on the tic douloureux 367
Fox's, Mr. lectures announced 94
Fracaftorius on epidemics 209
Fracture, cafe of 445
——— of the fkull, cafe of 295
——— of the cranium, on 526
Frampton's, Mr lectures announced 95
France, vaccination in 419
Freake, Mr. on the ufe of humulus lupulus 432
Frogs, experiments on 84
Fulwood's Rents, on the cafe in 53
Fumigations recommended in epidemics 113
Fungus hæmatodes, on the 7

G.

Galvanifm, review of Wilkinfon's elements of 82, 371
——— medical application of 86
Galvanometer, defcription of 372
Gangrene, on white 387
Gafes mix with the atmofphere 108
Gaftrodynia, on 132
——— Mr. Fogo on 207
Geoghegan, Mr. on bubo 435
Gibbon on fcepticifm 150
Gibraltar, account of the malignant fever at 193
Girdleftone, Dr. on the food of infants 298
Girtanner, Dr. on the venereal difeafe 89
Glans, of ulcers on the 386
Goddard, Mr. on fractures of the cranium 526
Gold-leaf, effect of galvanifm on 372
Goldfon's, Mr. cafes, remarks on 4
——— Dr. Walker on 133
——— reviewed 268
Gonorrhœa, on 321
Goulard's treatife on lead, remarks on 498
Gout, Meff. Taynton and Scott on cold water in 141
——— anfwer to Dr. Scott on 112
——— on the ufe of humulus lupulus in 432
——— Mr. O'Neale's cafe of 228
——— review of Mr. Edlin's cafes of 274
——— Dr. Lowry on 349
——— remarks on 355
——— Dr. Kinglake on Mr. Mace's cafe of 357
——— effect of galvanifm in 375
——— Mr. Mantal, on the cooling treatment of 487
——— Dr. Kinglake's reply to Mr. Edlin on 460
——— on Sydenham's treatment of 561
Greafe in horfes, on the 191
Grotto del' Cane in Italy, defcription of 108
Guy's hofpital, lectures at 94
——— improvements at 95

H.

Hæmatemefis, cafe of 239
Hæmorrhage, on 241
Haighton's, Dr. lectures announced 94
Hall, Dr. on Catarrh 209
Harmattan wind fufpends inoculation 1
Harrifon, Mr. on inoculation 331
Head, on ring-worm of the 24
Head-ach, effects of fal ammoniac and vinegar in certain cafes of 510
Headington's Mr. lectures announced 95
Health and longevity, on 528
——— committees of recommended 273
——— rules for preferving 531
Healthy, advice to the 116
Hector, Mr. on vaccination 52
Heart, cafe of mal-formation of the 120, 384
——— Dr. Walker's obfervations on the 429
Hebberden, Dr. on the food of children 298
Hemicrania, on 369
Hemiplegia, on galvanifm in 373
Hernia, effects of fal ammoniac and vinegar in ftrangulated 509
Herpetic eruptions, on 415, 541
Hey, Mr. reply to 7
Higgins's, Dr. meteorological table 185, 384, 480
Highlanders, on difeafes of the 28
Hill, Mr. on cutaneous eruptions 537
Hippocrates on epidemics 209
Home's obfervations on cancer, reviewed 361
Hope, Mr. on hydrophobia 297
Horn, Mr. on vaccination 59
Horfes, of the greafe in 191
——— on running 536
Hot climates, on difeafes of 481
Houfes in the Highlands defcribed 33
Howard's, Mr. cafe of fpina bifida 496
Howifon, Dr. receives a gold medal from the fociety of arts 383
Hugo, Mr. on vaccination 49
Humboldt on the medical application of galvanifm 86
Hume, Mr. on modern pharmacopœias 217

Humerus, on luxations of 528
Humulus lupulus, on the ufe of 432
Hungarian fever, of the 117
Hunter, Mr. on a cafe of fmall pox and the flough 6
Hurricanes, on the origin of 110
Hydatids of the breaft, cafes of 365
Hydrocele, on the 389
——— effects of fal ammoniac in 509
Hydrogen, water from oxygen and 575
Hydrophobia, cafe of 155
——— on cupping in the cure of 242
——— receipt for 297
Hyofcyamus, its efficacy 477

I. J.

Icelandic mofs found in Italy 383
Ietros, anfwer to 258
Ignis fatuus, on the 111
Impediments of fpeech, on the 450
India, ftate of vaccination in 283, 336, 381
Indians, North American, bathing ufed by 522
Infants, on the food of 298
Inflammable bodies, how to preferve them from fire 288
Inflammatory affections, cure for 549
——— complaints, on 376
Influenza, on the 213
Influenza of China, on the 487
Inglis's, Dr. cafes of difeafed tongue 179
Inoculation fufpended by the Harmattan wind 1
——— obfervations on 165
——— Mr. Ring on 168, 245, 570
——— ftatements of vaccine 188
——— Mr. Harrifon on 331
——— Mr. Forbes on 517
Invalid, or means of enjoying health and long life, reviewed 81
Ireland, vaccine inftitution in 379
Itch, caufes of in the highlands 35
Jackfon, Dr. on the conftitution of the medical department of the Britifh army, reviewed 275
——— on the treatment of fevers 472, 564
Jenkinfon, Mr. on folutio arfenici in rheumatifm 525
Jenner, Dr. account of a buft of 575
———'s, diploma from the univerfity of Wilna 428
———'s letter to Mr. Shoolbred 569
Jennerian Society, communications to the Royal 94, 283, 379, 479
——— ——— account of the Royal Somerfet 187
——— ——— at Plymouth Dock, refolutions of 91
——— on herpetic eruptions 541
Jockies, on training 536

K.

Kentifh, Dr. on the treatment of burns 14
——— on the ufe of bark in gout 350
King's, Dr. obfervations on inoculation 165
——— ——— on fcarlatina anginofa 167
Kinglake's, Dr. remarks on Mr. O'Neal's cafe 231
——— on Mr. Mace's cafe of gout 357
——— reply to Mr. Edlin's two cafes, reviewed 460
——— reply to Dr. Scott, on gout 512
Kinning's, Mr. account of the malignant fever at Gibraltar 193
Krafkovitz's, Dr. letter to the Royal Jennerian Society 479

L.

Larrey's cafes, obfervations on Mr. 150
Lawrence's, Mr. treatife on cattle, reviewed 564
Lazarettos, floating ones recommended 273
Lead, its effects as a fedative 498
LECTURES announced: at St. Thomas's and Guy's Hofpitals, 94. Mr. Cline and Mr. Cooper's on Anatomy and Surgery, *ib.* Dr. Babington and Dr. Curry on the Practice of Medicine, *ib.* Dr. Babington and Mr. Allen on Chemiftry and Experimental Philofophy, *ib.* Dr. Curry on the Theory of Medicine and Materia Medica, *ib.* Dr. Haighton on Midwifery and the Difeafes of Women and Children, and on Phyfiology, *ib.* Drs. Babington, Curry, and Marcet's Clinical, *ib.* Mr. Fox on the Teeth, *ib.* Mr. Coleman on the Veterinary Art, *ib.* Medical Theatre, St. Bartholomew's Hofpital. Dr. Roberts and Dr. Powell on the Theory and Practice of Medicine, 95. Mr. Abernethy on Anatomy, Phyfiology, and Surgery, *ib.* Mr. Macartney on Comparative Anatomy, and the Laws of Organic Exiftence, *ib.* Dr. Powell and Dr. Edwards on Chemiftry and the Materia Medica, *ib.* Dr. Thynne on Midwifery and the Difeafes of Women and Children, *ib.* Theatre, London Hofpital, Mr. Headington and Mr. Frampton on Anatomy, Phyfiology, and Surgery, *ib.* Mr. Armiger's Demonftrations and Diffections, *ib.* Dr. Dennifon on Midwifery, *ib* Dr. Badham's, on Medicine and Chemiftry, 96. Mr. Brookes's, on Anatomy, Phyfiology, and Surgery, *ib.* Dr. Bradley's, on the Practice of Medicine, *ib.* Dr. Batty's, on Midwifery, *ib.* and 288. Mr. Blair's, on Phyfiology, *ib.* Mr. Chevalier's, on Surgery, *ib.* Mr. Milburne's, on Phyfiology, 191. Mr. Moor's, on the Teeth, *ib.* Mr. Taunton's, on Anatomy, Phyfiology, and Surgery, 479. Dr. Reid on the Theory and Practice of Medicine, 576.

Leeches, on the management of 76

——— Dr. Fergufon on 299

Legs, how to reduce œdematous 288

Lepra, on bliſters in 140

Leproſy, on Moſes's deſcription of the 36

Ligature, by whom invented 391

Limb, on binding up an amputated 101

Lime, perſons dying in epidemics ſhould be buried in 116

Lind's, Dr. letter to Dr. Batty 574

Liſbon, dirty ſtate of 118

London, account of diſeaſes in an eaſtern diſtrict of 89, 186, 282

——— hoſpital, lectures at 95

Longevity, on health and 528

——— rules for promoting 531

Low, Mr. on ring-worm of the head 24

Lowry, Dr. on gout 349

Lubbock's, Dr. caſe of catalepſy 180

Lumbago, effects of ſolutio arſenici in 525

Lungs, on ulcerations of the 152

Luſcombe's, Mr. caſes of epidemic fever 348

Luxations of the humerus, on 528

Luxury, on 81

M.

Macartney's, Mr. lectures announced 95

Macdonald's, Dr. paper, obſervations on 3

M'Mullin, Mr. on the treatment of the chorea ſancti viti 178

Mace's caſe of gout, remarks on 357

Mad dog, receipt for the bite of a 297

Male, Dr. on an extraordinary appearance of the ſtomach 164

Mal-formation, caſe of 120

——— remarks on 429

Mangrove, tan made from the bark of the 383

Manmidwife, impropriety of the term 190

Manning's, Mr. buſt of Dr. Jenner, account of 575

Mantal, Mr. on Mr. Baker's caſe 144

——— on the cooling treatment of gout 437

Marcet's, Dr. lectures announced 94

Mead, Dr. on his medical character 137

Medical and phyſical intelligence, 89, 186, 283, 379, 478, 574

——— lectures, ſee LECTURES

Medicine, on the application of galvaniſm to 85

Mercury, on its application in venereal affections 395

——— its efficacy in ſyphilis 79

——— in venereal affections, on 549

Metal, diſcovery of a new 90

Meteorological table at Brompton, 185, 384, 480

Milburne's, Mr. lectures announced 191

Milne's, Dr. botanical dictionary, reviewed 564

Mineral acids, on 112

Mirza Mehedy Ali Khan on vaccination 574

Mollities oſſium, caſe of 392

Moor's, Mr. lectures announced 191

Mortality, bill of, for Portſmouth in New Hampſhire 92

Mortifications, on nitre in 140

Moſeley's, Dr. treatiſe on lues bovilla, reviewed 466

Moſes, accuracy of his deſcription of the leproſy 36

Muriat of antimony, on 447

Muſcles, on galvaniſm in involuntary action of the 374

Muſtard, compoſition of the eſſence of 502

N.

Natural hiſtory, curious fact in 287

Nerves, on galvaniſm in diſorders of the 86

Nervous diſeaſes, on 477

Net-retractor, on the 100

Nitre, its uſe in ſphacelus 324

——— on its application in mortifications 140

Nitrous acid, on its uſe in ſyphilis 79

——— its efficacy in diabetes mellitus 152

Noehden's, Dr. death announced 480

Nomenclature, on medical 227

Nonagenarian's invalid, or means of enjoying health, reviewed 81

North, the Hon Mr to Dr. de Carro 128

Noſology, on forming a ſyſtem of 560

O.

Œdematous legs, how to reduce 288

Oil, on its uſe in yellow fever 17, 108

——— compoſition of Britiſh 502

O'Neal's, Mr. caſe of gout 228

——— Dr. Kinglake's remarks on 231

Opiates, on, in catarrh 216

Opium adminiſtered in caſes of amputation 102

——— on purified and crude 226

Oſcheocele, caſe of 401

Oſſifications, on pulmonary 385

——— on tendinous, *ib.*

Oxyde of lead, on 223

Oxyds, on metallic 373

Oxygen, its efficacy in ſyphilis 78

——— on the want of it in the human body 106

——— water from hydrogen and 575

P.

Palmer's, Prof. compoſition for preſerving inflammable bodies 288

Paralytic affections, efficacy of Galvaniſm in 374

Pareé not the inventor of the ligature 391

Peaal, Mr. on the diſeaſes of the Highlanders 28

Pearſon's, Dr. Plan to prevent Contagion, reviewed 272

Pearfon, Mr. on difeafes of hot climates 481
———, on the influenza in China 487
———'s cafe of diffection 490
Peat fires encourage the itch 36
Pelvis, cafes of tumours in the 178
Peftilential difeafes, on the caufes of 211
Pharmacopœias, on modern 217
Phthifis pulmonalis, cafe of 323
Phymofis, on the application of fal ammoniac and vinegar in 511
Pitt's, Mr. cafe of fracture 445
Plague, on the 107
Platina, new metal difcovered in crude 90
———, amalgam of, defends copper veffels from acids 288
Platt, Mr. in reply to Mr. Whately 548
——— on oxygen in fyphilis, reviewed 78
Plymouth Dock, resolutions of the Jennerian Society at 91
Pontine marfhes, on the 118
Poor, activity of the fociety for bettering the condition of the 190
Porter, importance of genuine 115
Portfmouth, (New Hampfhire) bill of mortality at 92
Poftmafters General, their liberality 94
Powell's, Dr. lectures announced 95
Prepuce, on ulcers on the 386
Procidentia uteri, on 129
Pugilifts, on training of 532
Pulmonary offifications, on 386
Pythagoras, obfervation of 82

Q.

Quarantine, defects in the fyftem of 117
Queries relating to fyphilis 89
——— fcarlatina 167
——— malignant fevers in the metropolis 190
——— on athletic exercifes 532

R.

Rabies canina, cafe of 155
———, recipe for 297
———, on the fpontaneous origin of 478
Reid's, Dr. lectures announced 576
——— on the warm baths of Ruffia 77
——— on confumption, announced 479
Re-inoculation, cafe of 38
Retractor, advantages of the net 100
Rheumatifm, on folutio arfenici in chronic 16, 525
Rhodium, a new metal, account of 90
Rickerton's, Dr. means of preferving health, announced 288
Riga balfam, compofition of 502, *note*
Ring, Mr. on Dr. Coxe's cafes 48
——— his cafe of malformation of the heart 120
——— in reply to Dr. Adams 168
——— on inoculation 170, 245, 570
——— on Mr. Shoolbred's report 336
Ring, Mr. his communication of Chaptal's letter 419
——— remarks on his cafe of malformation 429
——— on vaccination 457
Ring-worm of the head, on 24
Roberts's, Dr. lectures announced 95
Rogers, Mr. on the evidence relative to the cow-pox, reviewed 468
Running horfes, on training 536
Rupture, cafe of uterine 234
Rufh's, Dr. Elements of life, announced 288
Rumfey's, Mr. cafe of enteritis 182
Ruffia, on the warm baths of 77
———, volcanic eruption in 110
Ruffians, on the conftitution of the 522
Rutty's, Dr. account of a difeafe among horfes 216, *note*
Ryal, Mr. on cold affufion in colic 18

S.

Sailors, their inferiority as boxers, &c. 532
Sanders's, Mr. propofal for a difpenfary for difeafes of the eye and ear 96
Sarco-epiplocele, on 291
Sarcoma, on the adipofe 388
Scarlatina anginofa, on 167
Sciatica, effects of fal ammoniac in vinegar, on 509
———, effects of folutio arfenici in 525
Scirrhus tefticle, on 293
Scorbutic affections, on humulus lupulus in 433
Scott, Dr. on Mr. Cuming's reply to 135
——— Dr. Kinglake's anfwer to 512
Scott, Mr. on cold water in gout 141
Scrofula, effects of Galvanifm in 375
Shearly's, Mr. cafe of abfcefs in the cheft 21
Shoolbred's, Mr. letter to Dr. Jenner 569
——— Report of vaccination in Bengal 257
——— Mr. Ring's remarks on 336
Simmons's, Mr. reply to Mr. Hey 7
———, felect fubjects in furgery 7, 97, 289, 385
Simpfon, Mr. on ofcheocele 491
——— on fphacelus 324
Sinclair, Sir John, on health and longevity 192, 528
Sivvens, on the difeafe called 89
Slough, on the 6, 400
Skull, cafe of fractured 295
Small-pox, cafe of a child born with 6
———, decreafe of deaths in 189
———, fubfequent to vaccination, on 268
Smoking, on 34
Snake, cow fucked by a 287
Snow's, Mr. cafe of fractured fkull 295
Soda, fulphat of, its efficacy in bubo 38
Somerfet Jennerian Society, account of 187
Spain, on the epidemic fever in 103
———, French phyficians fent to 383

Spalding's, Dr. bill of mortality for Portsmouth (New Hampfhire) 92
Specific, on the word 138
Speech, on impediments of 450
Spens's, Dr. cafes of erythema mercuriale 177
Sphacelus, on nitre in 324
Spina bifida, cafe of 496
Sponge, its efficacy in bronchocele 13
Sprains, effects of fal ammoniac in vinegar in 508
Squirrell, Dr. on the cow-pox, reviewed 465
Steatoma, on 289
Stock's, Dr. collections on cold as a remedy 192
Syphilis, on oxygen in 78
——, queries on the hiftory of 79
Stomach, extraordinary appearance of difeafed 164
Stott, Mr. on muriat of antimony 447
Straufs, M. on the action of acids 288
Subterraneous fires, on 111
Surgery, on felect fubjects in 7, 97, 289, 385,
Surgical cafes, on fal ammoniac in vinegar in 497
Sydenham, on difeafe 81
——, on the atmofphere in producing difeafe 209
Syer's, Mr. cafe of hæmatemefis 239

T.

Tan made from the bark of the mangrove 383
Tartrite of antimony, experiments on 447
Taunton's, Mr. lectures announced 479
—— on the baths of Ruffia 77
Taynton, Mr. on cold water in gout 141
Teething of children, on 516
Temperance, on 81
Tendinous offifications, on 385
medy, announced 192
Tendo Achillis, cafe of ruptured 494
Teftes, effects of fal ammoniac and vinegar in tumefaction of the 509
Tefticle, on fcirrhus 293
Thelwall, Mr. on impediments of fpeech 450
Thomas's, St. hofpital, lectures at 94
Thompfon, Mr. on vaccination 51
Thornton, Dr. on vaccination, announced 192
Thynne's, Dr lectures, announced 95
Tic douloureux, account of the 367
Tinea capitis, obfervations on 8
Tongue, cafes of difeafed 179
Tourniquet, of the 98
Trotter, Dr. on gaftrodynia 132
——, Mr. Fogo in anfwer to 207
——, his cafe of mollities offium 392
Tumours of the pelvis, cafes of 178
Turkey, progrefs of vaccination in 479

U.

Ulcerations of the lungs, on 152
Ulcers, of the glans and prepuce 386
—— of the genitals, on the 79
Urethra, on ulcerations in the 321
——, on ulcers in the 548
Urinary bladder, on the puncture of the 10
—— organs of a child, cafe of 179
—— on malformation of ib.
Uteri, on procidentia 129
Uterine rupture, cafe of 234
Uterus, on an enlargement of the 387

V.

Vaccination, Dr. Adams on 1
——, progrefs in the Highlands 37
——, Dr. Coxe on 4●
——, Mr. Ring on 48, 457
——, Mr. Hugo on 49
——, Mr. Thompfon on 51
——, Mr. Hector on 52
——, Dr. Wood on 57
——, Mr Horn on 60
——, Mr. Chriftie on 122
——, cafes of fmall-pox fubfequent to 268
——, Mr. Dunning on 418
——, Chaptal's letter to the Prefects on 420
——, Dr. Walker on 455
——, Mr. Shoolbred on 569
Vaccine inoculation, ftatement of 188
—— in Bengal, report on 257, 283, 381
—— Inftitution at Edinburgh 286
—— and variolous inoculation, cafes of 517
Vacciola, on 65
Van Marum, Galvanic experiment by 373
Vapour bath, on 522
Venereal affections, on mercury in 395, 549
—— difeafe, queries on the natural hiftory of the 79
Verdigris, on 224
Vefuvius, on the eruption of, in 1794 109
Vinegar, on fal ammoniac in 497
Vitriol, on white, in agues 138

W.

Waiblinger's, Mr. cafe of ruptured tendo Achillis 494

Walker, Dr. on the Fullwood's Rents cafe 53

——, on vaccine matter 94

——, on Mr. Goldfon's pamphlet 183

——, Mr. Creafer's letter to 186

——, Dr. Anderfon's letters to 283

——, on Mr. Ring's cafe of malformation 429

——, on vaccination 455

——, his anfwer to Eofaco 539

——, Mr. Dunning in reply to 542

Water formed from hydrogen and oxygen 575
——, on cold, in gout 141, 144, 228, 231, 274, 355, 357, 437, 460
——, in colic 18
——, on the action of Galvanifm in the decompofition of 373
——, in fprains, on its efficacy 500
Whately, Mr. on ulcerations in the urethra 321
——, on luxations of the humerus 520
——, reply to 548
White gangrene, on 387
Whitlam, Mr. on folutio arfenici in rheumatifm 16
——, on the management of leeches 76
Wilkinfon's, Mr. reply to Mr. Cuming 135
——, Mr. Cuming in reply to 325
——, Elements of Galvanifm reviewed 82, 371
——, on fal ammoniac in vinegar 497
Wilna, diploma from the univerfity of, to Dr. Jenner 428
Williams, Mr. on oil in yellow fever 17
Wilfon, Dr. on febrile difeafes, reviewed 554
Wollafton's, Dr. account of a new metal 90
Wood, Dr. on vaccination 57
Woodham, Mr. on Mr. Larrey's cafes 150
Worms, on cowhage in difeafes occafioned by 87
Wounds, on the application of fal ammoniac and vinegar in lacerated 507
Wreftlers, on training 532

Y.

Yaws, on the 79, 90
Yellow fever, on the ufe of oil in the cure of 17, 108
——, account of the, at Catfkill, North America 304
——, not contagious 378
——, obfervations on 107
Young's, Mr. work on cancer, announced 383

### REFERENCES TO THE PLATES.

Mr. Simmons's New Surgical Instruments — — 100
Dr. Male's Case of diseased Stomach — — — *ib.*
Mr. Ring's Case of Malformation of the Heart — — 120
Mr. Simmons's Case of steatomatous Tumour — — 289
—— open Cancer — — — 294
—— scrofulous Testis — — 293
—— pulmonary Ossifications and schirrhus Testicle — — — — — — — — 386
Mr. Waiblinger's Case of ruptured Tendo Achillis — 495
Mr. Howard's Case of Spina Bifida — — — *ib.*

